# Exploring Latent Profiles of Metacognition and Their Impact on Computational Thinking and Mathematical Modeling Competency in High School Students

**DOI:** 10.3390/jintelligence14070138

**Published:** 2026-07-03

**Authors:** Yu Zhou, Bin Jing, Jing Zhang

**Affiliations:** 1 Faculty of Education, Shaanxi Normal University, South Chang’an Road 199, Yanta District, Xi’an 710062, China; zhouyu@snnu.edu.cn (Y.Z.); jingbin@snnu.edu.cn (B.J.); 2 School of Mathematics and Data Science, Changji University (New Campus), No. 9 South Shiji Avenue, Changji City 831100, China

**Keywords:** metacognition, computational thinking, mathematical modeling competency, latent profile analysis

## Abstract

As the capacity to monitor and regulate one’s own cognitive processes, metacognition is considered pivotal for mathematics learning and the development of higher-order thinking skills. However, research from a person-centered perspective remains limited regarding individual differences in metacognition and their associations with mathematics-related abilities. To address this gap, the present study surveyed 512 high school students and employed Latent Profile Analysis (LPA) to identify three distinct metacognitive profiles: “High metacognition,” “Medium metacognition,” and “Low metacognition,” accounting for 17.96%, 53.52%, and 28.52% of the sample, respectively. The BCH method revealed significant between-profile differences in computational thinking and mathematical modeling competency, with the “High metacognition” group demonstrating the strongest performance. Mediation analysis indicated that computational thinking partially mediated the relationship between metacognitive profiles and mathematical modeling competency, with the effect being most pronounced in the “High metacognition” group. These findings suggest substantial heterogeneity in students’ metacognition and highlight the potential relevance of prioritizing metacognitive cultivation in mathematics education. They further suggest that fostering computational thinking may be a productive route to strengthening mathematical modeling competency.

## 1. Introduction

In recent years, metacognition, which refers to the capacity to understand, monitor, and regulate one’s own cognitive processes (Flavell, 1979), has garnered increasing research attention in both psychology and mathematics education (Okumuş & Öztürk, 2024; Schneider & Artelt, 2010). It is widely recognized as a cornerstone of self-regulated learning, providing a fundamental mechanism that enables learners to identify their cognitive characteristics and adapt strategies to enhance problem-solving performance (Li et al., 2023; Schraw & Moshman, 1995; Zimmerman, 2008). This regulatory function is particularly important in mathematics education. Although the field places strong theoretical emphasis on higher-order thinking, students often experience mathematics as procedurally oriented. In such contexts, self-regulation supports the transition from procedural execution to deeper mathematical reasoning (Wijaya et al., 2015).

According to the theory of self-regulated learning, metacognitive competence enables learners to actively adjust their thinking and behavior, thereby exerting a positive influence on their problem-solving processes (Zimmerman, 2008; Schneider & Artelt, 2010). Research has indicated that metacognition effectively promotes computational thinking (Üzümcü, 2023; Yadav et al., 2022). Specifically, it directly supports core computational thinking activities, such as decomposing problems, creating abstract models, and designing algorithms, by strengthening self-monitoring and evaluation during problem-solving (C. Y. Wang et al., 2024; Yadav et al., 2022). Furthermore, the systematic problem representation and abstraction skills required by computational thinking lay the groundwork for tackling complex tasks (Ang, 2021; Wu & Yang, 2022). Concurrently, metacognition has been shown to significantly predict mathematical modeling competency (Duman & Aydoğan Yenmez, 2024; He et al., 2024; Hidayat et al., 2020). Throughout the modeling process, it enables students to consciously align strategies with goals and to refine their solutions through continuous iteration (He et al., 2024; Hidayat et al., 2018, 2023). Empirical evidence has consistently supported these positive correlations (e.g., Duman & Aydoğan Yenmez, 2024; He et al., 2024; Üzümcü, 2023). Building on these connections, researchers have proposed that computational thinking may mediate the effect of metacognition on mathematical modeling. Specifically, metacognition may enhance modeling performance by systematically cultivating an individual’s capacity for decomposition, abstraction, and algorithmic thinking (Zhang et al., 2024).

However, most existing research has adopted a variable-centered paradigm, focusing on the average relationships among metacognition, computational thinking, and mathematical modeling at the group level (He et al., 2024; Hidayat et al., 2023). This perspective overlooks the multidimensional nature of metacognition; consequently, learners may demonstrate qualitatively different patterns in how these dimensions interact, forming distinct metacognitive profiles (Schneider & Artelt, 2010; Yadav et al., 2022). Such profile-level differences, reflecting individuals’ overall cognitive configurations, may shape the development of computational thinking and mathematical modeling ability and the interplay between them (Bergman & Magnusson, 1997; Bergman & Trost, 2006). Neglecting this heterogeneity risks masking unique developmental pathways and underlying mechanisms that may exist across diverse subgroups of learners (Hidayat et al., 2023; Yadav et al., 2022).

To address this research gap, the present study adopts a person-centered perspective focusing on high school students, a developmental stage critical for the maturation of advanced cognitive abilities and higher-order thinking. Moving beyond an examination of aggregate variable-level relationships, this study aims to investigate the heterogeneous characteristics of metacognition and their specific associations with computational thinking and mathematical modeling ability.

## 2. Literature Review

### 2.1. Metacognition and Mathematical Modeling Competency

Metacognition is frequently defined as “cognition about cognition,” referring to an individual’s capacity to perceive, comprehend, and regulate their own cognitive activities (Flavell, 1979). Within the context of mathematics education, this concept manifests as students’ self-regulatory abilities during learning, encompassing an awareness of their cognitive strengths and strategic preferences, alongside the ability to plan, monitor, and adapt their thinking in response to task demands (Okumuş & Öztürk, 2024). Research has operationalized this construct through a three-dimensional framework in mathematics consisting of metacognitive knowledge, experience, and monitoring (G. Wang et al., 2017). Metacognitive knowledge involves an individual’s awareness of mathematical tasks, strategies, and the self (G. Wang et al., 2017; Zhang et al., 2024). Metacognitive experience denotes the affective and cognitive feedback generated during the learning process (Tay et al., 2024; Zhang et al., 2024). Meanwhile, metacognitive monitoring comprises the planning, oversight, evaluation, and reflection applied to the learning process itself (Cui et al., 2018; G. Wang et al., 2017). Crucially, this process extends beyond cognitive monitoring to incorporate the affective feedback characteristic of metacognitive experience (Tay et al., 2024; Zhang et al., 2024). Consequently, effective monitoring requires learners to regulate not only their strategic approach but also their emotional responses.

Mathematical modeling competency is a foundational requirement for the comprehensive application of mathematical knowledge to resolve complex real-world problems (Kaiser, 2007). This competency encapsulates an individual’s capacity to perform tasks and navigate the modeling cycle within diverse contexts (Cevikbas et al., 2022; He et al., 2024). Its essence lies in the abstraction of real-world situations into mathematical models, their subsequent analysis and resolution through mathematical methods, and the resulting explanation or prediction of reality (Galbraith et al., 2007; He et al., 2024). This process encompasses sub-competencies such as simplification, mathematization, interpretation, and verification (Kaiser, 2007; Cevikbas et al., 2022). Students with stronger modeling competency are better able to translate real-world problems into mathematical structures and construct viable solutions (Hankeln et al., 2019; He et al., 2024). Furthermore, active engagement in modeling activities fosters deeper conceptual understanding, cultivates higher-order thinking, and enhances integrative problem-solving capacity (Galbraith et al., 2007; Hidayat et al., 2023; Kaiser & Schwarz, 2006).

A theoretically grounded and empirically supported linkage exists between metacognition and mathematical modeling competency (Dinsmore et al., 2008; He et al., 2024; Zimmerman, 2008). From the perspective of self-regulated learning theory, successful engagement in mathematical modeling requires learners to deliberately coordinate cognitive and metacognitive resources, including goal setting, progress monitoring, pathway evaluation, and assumption revision. These processes are centrally enabled by adaptive adjustments informed by metacognitive monitoring and experience (Pan & Chang, 2025; Zimmerman, 2008). Within the modeling cycle, conscious access to metacognitive knowledge and experiences facilitates accurate problem conceptualization, critical appraisal of modeling assumptions, and flexible strategy selection (Krüger et al., 2020; Vorhölter, 2023). Empirical studies consistently report positive correlations between these two constructs (Hankeln et al., 2019; He et al., 2024; Hidayat et al., 2020, 2023). However, this relationship may not be uniformly consistent. Vorhölter (2023) observed that certain metacognitive strategies might exert limited promotional effects on specific modeling sub-capabilities, such as verification, suggesting that the underlying influence mechanisms possess distinct conditional and boundary characteristics. Consequently, further investigation into the intricate relationship between metacognition and mathematical modeling competency is warranted.

### 2.2. Computational Thinking as a Mediator

Computational thinking is increasingly recognized as a domain-transcendent cognitive paradigm that applies core computer science principles, including logical reasoning, algorithmic thinking, and pattern recognition, to problem-solving and system design (Ramírez-Echeverry et al., 2025; Wing, 2006, 2011). Although computational thinking is often operationalized through programming (Shute et al., 2017), its core cognitive dimensions, including abstraction, decomposition, and generalization, represent practices that extend well beyond computing (Selby & Woollard, 2013). In mathematics, problem-solving similarly requires identifying structural patterns, reducing complexity, and constructing generalizable procedures (Weintrop et al., 2016; Kong & Ming, 2026). Accordingly, computational thinking offers a coherent framework for analyzing mathematical problem solving, not merely a set of programming-related skills. Furthermore, its core dimensions encompass creativity, algorithmic thinking, critical thinking, collaborative skills, and comprehensive problem-solving abilities (Korkmaz et al., 2017).

Computational thinking and metacognition are closely and mutually reinforcing (Beyazhancer & Cepni, 2026; Yadav et al., 2022). Metacognition provides a critical high-level regulatory framework for computational thinking activities, as it facilitates the conscious planning of solution steps, the selection and application of appropriate strategies, and the continuous monitoring and evaluation of outcomes (Üzümcü, 2023; Zhang et al., 2024). This regulatory function significantly promotes the development of computational thinking and its multi-dimensional competencies (De Backer et al., 2022; García et al., 2016; Jiang et al., 2023; Joo & Park, 2023; Magno, 2010). Specifically, metacognitive monitoring optimizes problem decomposition and the clarification of procedural steps (Joo & Park, 2023), while enhanced strategic flexibility enables learners to dynamically adjust plans during execution (García et al., 2016; Sevgi & Karakaya, 2020). These processes effectively cultivate critical thinking and creativity (Jiang et al., 2023; Magno, 2010), while improving communication and co-regulation within collaborative contexts (De Backer et al., 2022; Zhang et al., 2024). Consequently, metacognition is recognized as a fundamental enabling factor for the advancement of computational thinking.

Furthermore, computational thinking offers a direct and systematic cognitive framework that supports the development of mathematical modeling competency (Aksoy et al., 2026; Lehmann, 2024; Yıldız & Poyraz, 2026). Mathematical modeling involves abstracting real-world situations into workable mathematical structures and interpreting results to validate predictions about the real world (Kaiser et al., 2006). Computational thinking and mathematical modeling are distinct but closely related constructs: learners may engage in elementary forms of modeling without explicit computational thinking awareness, yet competent engagement in the modeling cycle relies heavily on the procedural architecture computational thinking provides, particularly abstraction, decomposition, and algorithmic reasoning (Aksoy et al., 2026; Yıldız & Poyraz, 2026). Computational thinking is not synonymous with modeling nor with abstraction alone; rather, it provides a systematic toolkit that translates abstract modeling actions into structured, repeatable cognitive procedures (Lehmann, 2024). Specifically, abstraction and decomposition enable students to eliminate extraneous details and define the problem core (Ramírez-Echeverry et al., 2025); algorithmic thinking guides the construction of clear, repeatable solution procedures (Ang, 2021; Lehmann, 2024); and critical thinking ensures the rigorous verification and optimization of models during iterative phases (Kannadass et al., 2023; Zhang et al., 2024). Furthermore, creativity drives the generation of innovative solutions (Ang, 2021; Lu & Kaiser, 2022), while collaborative skills facilitate effective communication and collective knowledge construction in complex tasks (Korkmaz et al., 2017; Møgelvang & Nyléhn, 2023). Empirical evidence indicates that these dimensions of computational thinking significantly promote mathematical modeling capabilities (Ang, 2021; Wu & Yang, 2022). Consequently, computational thinking not only supports mathematical modeling but may also mediate the relationship between metacognition and modeling competency (Zhang et al., 2024). As a high-level self-regulatory capacity, metacognition may systematically shape and enhance an individual’s computational thinking skills, which then serve as structured cognitive tools applied to complex modeling tasks, ultimately leading to superior modeling performance.

### 2.3. The Need for a Person-Centered Approach to Examine Metacognitive Profiles

Most previous studies on metacognition have employed a variable-centered analytical approach, focusing on the average relationships between metacognition and outcome variables, such as computational thinking and mathematical modeling, at the aggregate group level (He et al., 2024; Hidayat et al., 2023; Li et al., 2024; Yadav et al., 2022). This paradigm implicitly assumes that the relationships between variables are homogeneous across individuals, thereby potentially masking substantial intra-learner variations (Bergman & Magnusson, 1997; Bergman, 2000; Bergman & Trost, 2006). In fact, metacognition is a multi-dimensional construct; different individuals may exhibit qualitatively distinct combinations across dimensions such as knowledge, experience, and monitoring, resulting in heterogeneous metacognitive profiles (Bergman & Magnusson, 1997; Schneider & Artelt, 2010). Research has demonstrated that these configurational differences can significantly influence problem-solving and academic achievement (Hidayat et al., 2023; Li et al., 2023), which, in turn, are associated with differential performance in mathematical modeling (Duman & Aydoğan Yenmez, 2024; Hidayat et al., 2020, 2023). Learners with advanced metacognitive profiles are able to more effectively identify key problem characteristics, analyze logical relationships, and develop systematic solutions. Such capabilities not only improve their programming skills but also further reinforce their computational thinking (Li et al., 2023; Yadav et al., 2022).

To address the limitations of variable-centered approaches that overlook individual differences, the person-centered perspective, particularly through Latent Profile Analysis (LPA), offers a more nuanced analytical framework (Bergman, 2000; Hickendorff et al., 2018). LPA enables researchers to identify latent subpopulations characterized by distinct combinations of metacognitive traits, derived from individual response patterns across multiple observed variables (Spurk et al., 2020; Vermunt, 2010). Rather than presupposing group homogeneity, this method aims to reveal naturally occurring heterogeneity within a population and facilitates the examination of variations in outcome variables, such as computational thinking and mathematical modeling competency, across different profiles (Hickendorff et al., 2018). Despite its advantages, few studies have yet utilized this approach to explore the heterogeneity of metacognition or its subsequent relationship with computational thinking and mathematical modeling performance. Adopting a person-centered perspective is therefore essential to examining how metacognitive profiles shape computational thinking and mathematical modeling competency across diverse learners.

## 3. The Present Study

**Figure 1:**
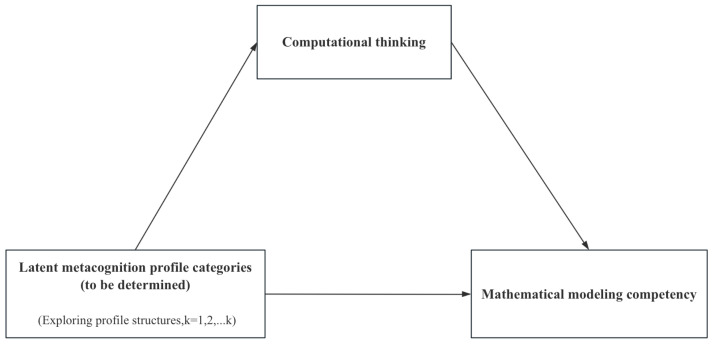
The hypothesized model.

Although variable-centered research has provided fundamental insights into the relationships among metacognition, computational thinking, and mathematical modeling ability, existing studies have generally overlooked the inherent individual heterogeneity of metacognition as a multi-dimensional construct. To address this critical limitation and considering that the high school stage is a pivotal period for the development of students’ cognitive and metacognitive capacities, this study adopts a person-centered analytical perspective. Rather than examining aggregate group averages, this approach aims to explore how metacognitive heterogeneity is differentially associated with computational thinking and mathematical modeling competency. Based on the theoretical model constructed for this investigation (as shown in Figure 1), this study addresses the following three research questions (RQs):

RQ1. What are the latent profiles of metacognition among high school students?
RQ2. Do significant differences exist in computational thinking and mathematical modeling competency across these distinct metacognitive profiles?
RQ3. What are the specific relationships between different metacognitive profiles, computational thinking, and mathematical modeling competency?

## 4. Method

### 4.1. Participants

This study was conducted in two provincial-level model high schools in City X, China, utilizing a sample comprising 12 classes across the 11th and 12th grades. A cluster sampling method was employed for sample selection. The two schools are comparable in terms of student entry-level achievement, instructional pace, and faculty resources, thereby reducing potential confounding at the school level. A total of 600 paper questionnaires and test papers were distributed. Students completed the computational thinking and metacognitive scales within 30 min, followed by a mathematical modeling competency test within 45 min.

To ensure data quality, rigorous inclusion criteria were applied. First, participants were excluded if their responses contained more than 20% unanswered items. For the remaining cases with less than 20% missing data, the Expectation-Maximization (EM) algorithm was utilized to impute missing values, ensuring the integrity of the dataset for subsequent analyses (Dempster et al., 1977). Second, participants were excluded if they exhibited clearly discernible invalid response patterns, defined as: (1) straight-lining (i.e., selecting the same response option for more than 90% of consecutive items within a scale); or (2) logical inconsistencies (e.g., providing contradictory answers to reverse-coded items) (DeSimone et al., 2015; Huang et al., 2012).

Following this process, 512 valid questionnaires were retained, yielding an effectiveness rate of 85.33%. Among the respondents, 260 were male (50.78%), and 252 were female (49.22%). The average age of participants was 17.04 ± 0.65 years. Additionally, 85 students (16.60%) were in the 11th grade, while 427 students (83.40%) were in the 12th grade.

### 4.2. Measures

#### 4.2.1. Metacognition

Metacognition was assessed using the Mathematical Metacognition Scale (G. Wang et al., 2017; see Appendix A for details), which comprises three dimensions: metacognitive knowledge (14 items), metacognitive experience (9 items), and metacognitive monitoring (27 items). Each item was rated on a 5-point Likert scale ranging from 1 (strongly disagree) to 5 (strongly agree), with higher scores reflecting greater metacognitive capacity. The scale’s respective dimensions exhibited robust reliability, as evidenced by Cronbach’s α coefficients of 0.80, 0.72, and 0.91 for the individual dimensions. In this study, the scale had suitable construct validity: χ^2^/df = 2.96, RMSEA = 0.06, CFI = 0.97, TLI = 0.97, SRMR = 0.03.

#### 4.2.2. Computational Thinking

The Computational Thinking Scale developed by Korkmaz et al. (2017) was used to measure students’ computational thinking dispositions, and this scale comprises five dimensions: creativity (4 items), algorithmic thinking (4 items), cooperativity (4 items), critical thinking (4 items), and problem solving (6 items). All items were rated on a 5-point Likert scale ranging from 1 (strongly disagree) to 5 (strongly agree). Higher scores reflect more positive self-perceptions of competence across these dimensions. The scale demonstrated acceptable to good reliability, with Cronbach’s α coefficients of 0.66, 0.72, 0.87, 0.79, and 0.80 for the respective individual dimensions. The structural validity of the scale was satisfactory: χ^2^/df = 2.70, CFI = 0.95, TLI = 0.92, RMSEA = 0.05, SRMR = 0.02.

#### 4.2.3. Mathematical Modeling Competency

Mathematical modeling competency was evaluated using a modeling test adapted from established high school-level tasks, which has been recognized as a reliable measurement tool (Hidayat et al., 2022). The assessment focuses on fundamental mathematical knowledge, strategic thinking, and practical application. It consists of three specific tasks: the shoe size problem, the revolving door problem, and the refueling problem (adapted from Zhang et al., 2024; see Appendix B for details). Each task is scored on a 5-point scale, resulting in a maximum possible score of 15. The test demonstrated acceptable internal consistency, with a Cronbach’s α coefficient of 0.78.

### 4.3. Data Analysis

All participants provided informed consent before data collection commenced. Data were gathered using paper-and-pencil testing methods. Following data collection, statistical analyses were performed in four stages using SPSS 27.0 and Mplus 8.3. First, SPSS 27.0 was utilized for descriptive and correlation analyses. Second, LPA was conducted with Mplus 8.3 to identify distinct metacognitive patterns. Model fit was assessed using the Akaike Information Criterion (AIC), Bayesian Information Criterion (BIC), and sample-size adjusted BIC (aBIC), where lower values indicate better model fit. Entropy values, ranging from 0 to 1, were evaluated to determine classification accuracy, with higher values indicating greater precision. Likelihood ratio tests, specifically the Lo-Mendell-Rubin (LMR) test and the Bootstrap Likelihood Ratio Test (BLRT), were applied. Significant *p*-values (*p* < 0.05) indicate that a k-profile model provides a significant improvement over a k-1 profile model. Additionally, each profile was required to include at least 5% of the total sample to ensure validity and representativeness (Lo et al., 2001; Nylund, 2007). Third, the Bolck-Croon-Hagenaars (BCH) procedure was implemented in Mplus 8.3 to examine differences in computational thinking and mathematical modeling competency across the identified profiles. Finally, the PROCESS macro (Model 4) for SPSS was employed to investigate the mediating role of computational thinking in the relationship between metacognitive profiles and mathematical modeling competency.

## 5. Results

### 5.1. Descriptive and Correlation Analysis

The descriptive and correlation analysis presented in Table 1 illustrates the relationships among metacognition, computational thinking, and mathematical modeling competency. The results indicate that the various dimensions of metacognition are significantly and positively correlated with both the various dimensions of computational thinking (0.05 < all r < 0.60, all *p* < 0.01) and mathematical modeling competency (0.20 < all r < 0.40, all *p* < 0.01), suggesting that higher metacognition is associated with stronger computational thinking dispositions and higher modeling scores. Additionally, the various dimensions of computational thinking and mathematical modeling competency exhibit a significant positive correlation (0.20 < all *r* < 0.40, all *p* < 0.01), indicating that stronger computational thinking dispositions are linked to success in mathematical modeling. In addition, Table 2 presents the descriptive statistics and independent-samples t-test results regarding gender and grade level for the various dimensions of metacognition and computational thinking, as well as for mathematical modeling competency. The results indicate significant differences based on gender and grade level in some dimensions of metacognition and computational thinking, as well as in mathematical modeling competency.

### 5.2. Latent Profile Analysis of Metacognition

LPA was employed to categorize metacognitive profiles within the sample. Table 3 presents the model parameters for each profile, while the fitting indices for one- to four-profile models were compared. The results indicated that AIC, BIC, and aBIC values consistently decreased as the number of profiles increased. The two- to four-profile models all demonstrated high entropy values exceeding 0.80, indicating robust classification quality. The LMR test confirmed that the three-profile model was superior to the two-profile model (*p* < 0.001), whereas the four-profile model did not yield a significant improvement over the three-profile model (*p* = 0.291). This suggests that the four-profile model did not offer a substantially better fit, particularly when considering the principles of parsimony and interpretability. Consequently, the three-profile model was identified as the optimal solution. Table 3 displays the mean scores of the three dimensions across the identified metacognition profiles.

**Figure 2:**
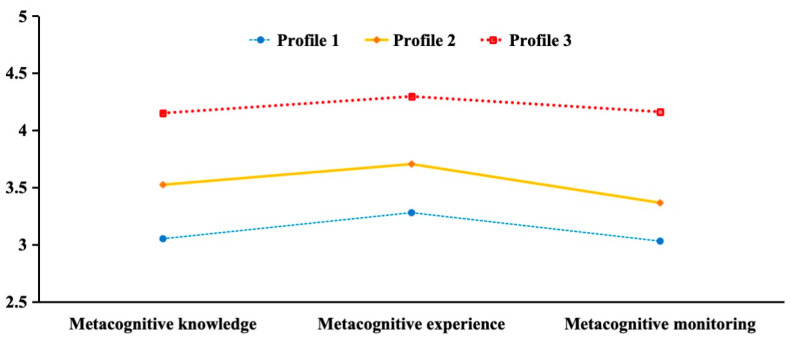
Three potential profiles of metacognition.

As illustrated in Figure 2, the scores across the three dimensions of metacognition, specifically metacognitive knowledge, experience, and monitoring, demonstrate a relatively consistent trend. This pattern suggests that these dimensions are closely interrelated; specifically, elevated levels in one dimension tend to be accompanied by high levels in the others. The Latent Profile Analysis (LPA) results reveal that 28.52% of students belong to Profile 1, 53.52% to Profile 2, and 17.96% to Profile 3, reflecting distinct levels of metacognitive competency across the student population. These findings underscore the interconnected nature of metacognitive dimensions and reveal meaningful heterogeneity in students’ overall metacognitive levels.

### 5.3. The Differences in Computational Thinking and Mathematical Modeling Competency in the Identified Profiles

The BCH method was utilized to analyze differences in computational thinking and mathematical modeling competency across the three latent metacognitive profiles (Bolck et al., 2004). As presented in Table 4, significant differences emerged among the three profiles in terms of metacognition, computational thinking, and mathematical modeling competency (all *p* < 0.01). Post hoc comparisons further indicated that, with the exception of the cooperativity dimension, scores for all other indicators followed a consistent trend: Profile 1 < Profile 2 < Profile 3. These findings indicate that the three latent profiles represent a hierarchical progression in overall metacognitive intensity rather than distinct configural types. The profiles were categorized as “High metacognition” (Profile 3), “Moderate metacognition” (Profile 2), and “Low metacognition” (Profile 1) and demonstrated clear, uniform differences across all metacognitive dimensions.

### 5.4. The Relationships Between the Three Latent Profiles of Metacognition, Computational Thinking, and Mathematical Modeling Competency

**Figure 3:**
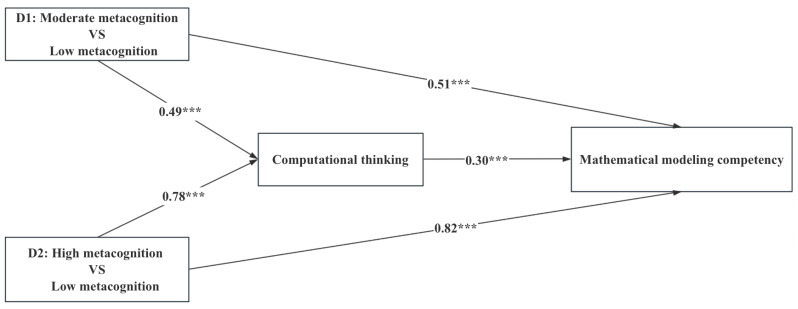
Mediation model. *n* = 512. *** *p* < 0.001. All data were standardized.

To examine the role of computational thinking in the association between metacognitive profiles and mathematical modeling competency, this study employed a mediation analysis. The analysis was conducted using the PROCESS macro for SPSS (Model 4) with 5000 bootstrap samples (Hayes, 2017). Metacognitive profiles were treated as the independent variable and were dummy-coded with “Low metacognition” as the reference group (D1: “Moderate metacognition” = 1; D2: “High metacognition” = 1). After controlling for gender, grade, and mathematics test scores, computational thinking was entered as a mediator and mathematical modeling competency as the dependent variable. The results revealed that, compared to the “Low metacognition” group, the “Moderate metacognition” group was associated with significantly higher levels of computational thinking (β = 0.49, *p* < 0.001) and mathematical modeling competency (β = 0.51, *p* < 0.001). Similarly, the “High metacognition” was associated with significantly higher scores on computational thinking (β = 0.78, *p* < 0.001) and mathematical modeling competency (β = 0.82, *p* < 0.001). Additionally, computational thinking was positively associated with mathematical modeling competency (β = 0.30, *p* < 0.001). A detailed diagram of the proposed pathway is provided in Figure 3.

The bootstrap test results for the indirect effect are summarized in Table 5. The 95% confidence intervals for the association between metacognitive profiles and mathematical modeling competency via computational thinking were [0.09, 0.21] (moderate vs. low) and [0.13, 0.34] (high vs. low). Since neither interval includes zero, a significant indirect effect is confirmed. This indicates that computational thinking accounts for a significant portion of the association between metacognitive profiles and mathematical modeling competency. Furthermore, the respective indirect effect sizes of 0.14 and 0.23 suggest that this indirect pathway is stronger for the High metacognition than for the Moderate metacognition.

## 6. Discussion

Using a person-centered approach, this study employed LPA to identify latent metacognitive profiles among the surveyed students and to examine variations in computational thinking and mathematical modeling competency across these groups. Furthermore, the investigation explored the relationship between diverse metacognitive profiles, computational thinking, and mathematical modeling competency. The following sections discuss these key findings and highlight their implications for both theory and educational practice.

### 6.1. Latent Profile Analysis of Metacognition

Through LPA, this study identified three distinct metacognitive profiles: “High” (17.96%), “Medium” (53.52%), and “Low” (28.52%). Although these profiles did not exhibit qualitatively different configural patterns (e.g., mismatched subscale levels), they address RQ1 by revealing meaningful heterogeneity in the overall strength of metacognition among high school students. This pattern suggests that metacognitive dimensions co-vary, functioning as a cohesive construct rather than as independent subtypes. This observed variation in overall intensity can be explained by several factors. Specifically, differences in individual cognitive development stages may lead to inherent variations in the awareness and regulation of one’s own learning processes (Chidley et al., 2025). Furthermore, disparate learning experiences and levels of prior training may enable certain students to become more adept at employing metacognitive strategies (Schneider & Artelt, 2010). Additionally, factors such as learning motivation, self-efficacy, and classroom instructional methodologies may further amplify individual differences in metacognitive development (Bozgün & Akın Kösterelioğlu, 2023). These findings highlight that individual differences in metacognition lie primarily on a continuum of overall intensity, rather than manifesting as discrete typologies. This provides a more nuanced framework for understanding individual differences in cognitive development and enriches the empirical evidence regarding metacognitive heterogeneity within specific regional or cultural contexts. These findings lay the groundwork for examining how metacognition interacts with computational thinking and mathematical modeling ability. They also suggest that educators should account for individual differences in metacognition when designing instructional strategies.

### 6.2. The Impact of Metacognitive Profiles on Computational Thinking and Mathematical Modeling Competency

Utilizing the BCH method, this study revealed that high school students across distinct metacognitive profiles exhibited significant inter-group differences in both computational thinking and mathematical modeling competency, thereby addressing RQ2. These findings support the premise that metacognition, acting as a higher-order cognitive regulatory system, plays a pivotal role in the cultivation of advanced competencies (Hidayat et al., 2023; Schneider & Artelt, 2010; Yadav et al., 2022). Specifically, the “High metacognition” profile demonstrated significantly higher scores in both domains. Consistent with prior research, this group reported higher levels of creativity, algorithmic thinking, critical thinking, and problem-solving (Herawaty et al., 2018; Jiang et al., 2023; Korkmaz et al., 2017; Magno, 2010). This was accompanied by a greater capacity to construct effective models aligned with real-world scenarios (Duman & Aydoğan Yenmez, 2024; Galbraith et al., 2007; Hankeln et al., 2019; Krüger et al., 2020).

In contrast, the “Medium metacognition” and “Low metacognition” groups exhibited comparatively lower scores in computational thinking and mathematical modeling competency. These score differentials likely stem from their constrained metacognitive knowledge and monitoring capabilities, which impede their conceptual understanding and the strategic application of mathematical principles. Furthermore, these limitations hinder their ability to translate abstract ideas into concrete models, thereby restricting their growth in these higher-order cognitive domains (Brown, 1987; He et al., 2024; Hidayat et al., 2018; Jiang et al., 2023). Notwithstanding these advantages, a nuanced pattern emerged regarding collaboration: students with high levels of metacognition demonstrated lower collaborative engagement than those with moderate levels. Drawing on the cognitive overload hypothesis, this seemingly paradoxical finding may be attributed to their superior metacognitive skills, which predispose them toward independent work. This preference can isolate high-metacognition students from diverse perspectives, which are themselves critical to the negotiation and refinement processes inherent in complex modeling tasks (Sweller, 1988; Kirschner et al., 2009). Accordingly, metacognitive training should be prioritized in educational settings, especially for students with lower metacognitive profiles, while incorporating collaborative learning structures to counteract the independent-work tendency observed among high-metacognition students. These findings illuminate how heterogeneous metacognitive profiles relate to advanced skill acquisition and lay an empirical foundation for investigating the complex relationship between metacognitive heterogeneity and higher-order cognitive abilities.

### 6.3. The Relationship Between Metacognitive Profiles, Computational Thinking, and Mathematical Modeling Competency

The mediation analysis suggests that computational thinking partially accounts for the association between metacognitive profiles and mathematical modeling competency, thereby addressing RQ3. Consistent with the proposed theoretical model, these results indicate that metacognitive profiles are both directly associated with modeling competency and indirectly linked via their association with computational thinking. These findings align with previous variable-centered research emphasizing concurrent associations among metacognition, computational thinking, and modeling competency; specifically, students with elevated metacognitive levels are more likely to cultivate robust computational thinking skills, thereby enhancing their mathematical modeling success (Hidayat et al., 2020; Vorhölter, 2023; Zhang et al., 2024). At the bivariate level, higher metacognition facilitates modeling competency through enhanced awareness, planning, self-examination, and the strategic deployment of cognitive tools, enabling students to analyze and resolve modeling tasks more effectively (Chaw & Tang, 2024; He et al., 2024; Hidayat et al., 2023). Furthermore, these findings extend the applicability of self-regulation theory (Dinsmore et al., 2008; Zimmerman, 2008), suggesting that metacognition strengthens self-regulatory capacities and heightens self-awareness. Such self-awareness helps students identify the knowledge needed for model construction. It also enables them to apply cognitive strategies—including logical reasoning, pattern recognition, and visualization—to analyze complex problems and arrive at systematic solutions (Brown, 1987; Dinsmore et al., 2008; Hidayat et al., 2018, 2020).

Moreover, distinct metacognitive profiles are associated with differential levels of computational thinking, and higher levels of computational thinking are significantly related to mathematical modeling performance. A plausible explanation for these findings is that high school students reporting robust metacognitive dispositions also tend to report superior planning, monitoring, and reflection capabilities, which coincide with more advanced levels of computational thinking (García et al., 2016; Joo & Park, 2023; Li et al., 2024; Sevgi & Karakaya, 2020; Yadav et al., 2022). Additionally, computational thinking encompasses analysis, data representation, and systematic modeling skills that are concurrently associated with mathematical modeling competency (Kayhan et al., 2024; Korkmaz et al., 2017). During the modeling process, the creative dimension of computational thinking aligns with the construction of diverse mathematical structures from multiple perspectives (Ang, 2021; Lu & Kaiser, 2022), while critical thinking correlates with the validity and accuracy of these models (Kannadass et al., 2023; Magno, 2010; Zhang et al., 2024). Correspondingly, algorithmic thinking and problem-solving abilities correspond to logical reasoning and practical application during the modeling phases (Lehmann, 2024; Korkmaz et al., 2017; Medová et al., 2019; Møgelvang & Nyléhn, 2023). These results reveal the cognitive pathways linking metacognition to mathematical modeling competency and offer a theoretical basis for fostering higher-order thinking in mathematics education.

## 7. Limitations and Future Directions

This study is subject to several limitations that should be acknowledged. First, computational thinking was assessed using a self-report disposition scale rather than a performance-based measure. Although such scales are widely used to gauge attitudes and self-efficacy in educational research, they are susceptible to individual biases and social desirability effects (Podsakoff et al., 2003). Consequently, the findings capture students’ self-reported dispositions rather than their demonstrated computational thinking proficiency. Similarly, metacognitive monitoring was also captured via self-report, which may not fully align with observed metacognitive behaviors. Future studies should triangulate self-reports with behavioral measures or teacher ratings for a fuller picture. Second, the cross-sectional design limits causal inference. While mediation relationships can be examined, directionality cannot be established. Given emerging evidence suggesting a potential bidirectional relationship between metacognition and computational thinking (Markandan et al., 2022; Román-González et al., 2017), longitudinal studies are needed to clarify the directionality and reciprocal dynamics of these relationships. Finally, the sample size and geographical scope constrain the generalizability of the findings. Future studies should recruit more diverse and larger samples across different educational systems and cultural contexts to strengthen the robustness and transferability of conclusions for mathematics education.

## 8. Conclusions and Implications

This study suggests significant individual differences in metacognition among high school students. It reveals three latent profiles, with the three profiles showing a clear hierarchical pattern in computational thinking and mathematical modeling competency. Mediation analysis suggests that computational thinking partially mediates the relationship between metacognitive profiles and mathematical modeling performance, with the effect being most pronounced for students in the high-metacognition group.

These findings suggest that educational practice should prioritize the inherent heterogeneity of metacognition by implementing differentiated instruction and personalized guidance. Educators should also leverage computational thinking as a bridge, designing integrated curricula that weave together metacognitive training, computational thinking, and mathematical modeling instruction. Concurrently, teachers should also leverage high-metacognition students as peer models and mentors, while designing tiered support strategies tailored to groups with distinct cognitive characteristics to systematically enhance modeling performance and higher-order problem-solving skills. For instance, establishing instructional guidance mechanisms based on cognitive profile classifications could provide appropriate learning scaffolds for diverse learners. Classroom instruction should explicitly incorporate training in problem decomposition, abstract representation, process monitoring, and reflective adjustment. Ultimately, by utilizing cooperative learning to harness the catalytic influence of cognitive leaders, a broadly applicable integrated instructional model can be developed to support all students.

## Figures and Tables

**Table 1:** Descriptive statistics and correlations.

	M ± SD	1	2	3	4	5	6	7	8
1 Metacognitive knowledge	3.50 ± 0.44								
2 Metacognitive experience	3.69 ± 0.45	0.68 **							
3 Metacognitive monitoring	3.41 ± 0.50	0.70 **	0.62 **						
4 Creativity	3.76 ± 0.63	0.20 **	0.22 **	0.21 **					
5 Algorithmic thinking	3.31 ± 0.64	0.43 **	0.36 **	0.36 **	0.42 **				
6 Cooperativity	3.57 ± 0.79	0.10 *	0.18 *	0.18 *	0.26 **	0.24 **			
7 Critical thinking	3.46 ± 0.67	0.29 **	0.32 **	0.27 **	0.48 **	0.56 **	0.23 **		
8 Problem-solving	3.27 ± 0.64	0.19 **	0.21 **	0.18 **	0.30 **	0.36 **	0.24 **	0.40 **	
9 Mathematical modeling competency	8.82 ± 3.68	0.34 **	0.30 **	0.25 **	0.21 **	0.31 **	0.16 **	0.33 **	0.31 **

Note: ** *p* < 0.01, and * *p* < 0.05.

**Table 2:** Descriptive statistics and independent-samples *t*-test results for the variables.

	Variables	M	SD	t	*p*
1 Metacognitive knowledge	Male	3.55	0.44	2.84 ***	*p* < 0.001
	Female	3.64	0.44		
	Grade11	3.53	0.44	0.69	*p* >0.05
	Grade12	3.49	0.44		
2 Metacognitive experience	Male	3.74	0.47	2.60 *	*p* < 0.05
	Female	3.64	0.44		
	Grade11	3.75	0.45	1.48	*p* >0.05
	Grade12	3.67	0.45		
3 Metacognitive monitoring	Male	3.46	0.50	2.25 *	*p* < 0.05
	Female	3.36	0.49		
	Grade11	3.43	0.49	0.37	*p* >0.05
	Grade12	3.41	0.50		
4 Creativity	Male	3.81	0.66	1.83	*p* >0.05
	Female	3.71	0.59		
	Grade11	3.73	0.57	−0.57	*p* >0.05
	Grade12	3.77	0.64		
5 Algorithmic thinking	Male	3.38	0.69	2.43 *	*p* < 0.05
	Female	3.24	0.57		
	Grade11	3.36	0.62	0.66	*p* >0.05
	Grade12	3.31	0.64		
6 Cooperativity	Male	3.61	0.85	1.27	*p* >0.05
	Female	3.52	0.73		
	Grade11	3.73	0.84	2.08 *	*p* < 0.05
	Grade12	3.54	0.78		
7 Critical thinking	Male	3.55	0.68	3.24 ***	*p* < 0.001
	Female	3.37	0.64		
	Grade11	3.52	0.65	0.84	*p* >0.05
	Grade12	3.45	0.67		
8 Problem-solving	Male	3.30	0.70	0.95	*p* >0.05
	Female	3.25	0.56		
	Grade11	3.35	0.55	1.16	*p* >0.05
	Grade12	3.26	0.65		
9 Mathematical modeling competency	Male	9.13	3.75	1.97 *	*p* < 0.05
	Female	8.49	3.59		
	Grade11	7.92	3.44	−2.50 *	*p* < 0.05
	Grade12	9.00	3.70		

Note: *** *p* < 0.001 and * *p* < 0.05.

**Table 3:** Fit indices for the four models using the LPA approach (*n* = 512).

Profile	AIC	BIC	aBIC	Entropy	LMR	BLRT	Group Size for Each Profile			
							1	2	3	4
1-profile	2010.67	2036.10	2017.05	-	-	-	512			
2-profile	1465.36	1507.75	1476.00	0.897	<0.001	<0.001	399	113		
3-profile	1308.31	1367.64	1323.20	0.802	<0.001	<0.001	146	274	92	
4-profile	1274.83	1351.12	1293.98	0.805	0.291	<0.001	140	264	75	33

**Table 4:** Descriptive statistics, BCH, and post hoc tests for the three profiles of metacognition on three-dimensional scores.

Variables	Profile 1 (146)	Profile 2 (274)	Profile 3 (92)	χ^2^	*p*	Post Hoc
Metacognitive knowledge	2.96 ± 0.03	3.57 ± 0.02	4.18 ± 0.02	1307.13	<0.001	1 < 2 < 3
Metacognitive experience	3.19 ± 0.03	3.75 ± 0.02	4.33 ± 0.03	716.83	<0.001	1 < 2 < 3
Metacognitive monitoring	2.97 ± 003	3.39 ± 0.02	4.20 ± 0.03	804.96	<0.001	1 < 2 < 3
**Metacognition**	**3.04 ± 0.02**	**3.57 ± 0.01**	**4.24 ± 0.02**	**18,844.02**	**<0.001**	**1 < 2 < 3**
Creativity	3.60 ± 0.05	3.80 ± 0.04	3.94 ± 0.09	14.58	0.001	1 < 2 < 3
Algorithmic thinking	2.99 ± 0.05	3.34 ± 0.04	3.76 ± 0.09	68.12	<0.001	1 < 2 < 3
Cooperativity	3.36 ± 0.06	3.71 ± 0.05	3.52 ± 0.11	16.19	<0.001	1 < 3 < 2
Critical thinking	3.24 ± 0.06	3.48 ± 0.04	3.78 ± 0.09	26.51	<0.001	1 < 2 < 3
Problem-solving	3.05 ± 0.05	3.34 ± 0.04	3.42 ± 0.10	24.19	<0.001	1 < 2 < 3
**Computational thinking**	**3.23 ± 0.03**	**3.52 ± 0.03**	**3.66 ± 0.06**	**55.11**	**<0.001**	**1 < 2 < 3**
**Mathematical modeling competency**	**6.52 ± 0.34**	**9.44 ± 0.24**	**10.81 ± 0.26**	**100.58**	**<0.001**	**1 < 2 < 3**

**Table 5:** The effect sizes of the mediating analysis.

	β (SE)	95% Confidence Interval	
		LLCI	ULCI
**Total effects**			
D1 → Mathematical modeling competency	0.65(0.09)	0.47	0.84
D2 → Mathematical modeling competency	1.05(0.13)	0.80	1.30
**Indirect effects**			
Indirect effect 1: D1 → Computational thinking →Mathematical modeling competency	0.14(0.03)	0.09	0.21
Indirect effect 2: D2 → Computational thinking →Mathematical modeling competency	0.23(0.05)	0.13	0.34
**Direct effects**			
D1 → Mathematical modeling competency	0.51(0.09)	0.33	0.69
D2 → Mathematical modeling competency	0.82(0.13)	0.58	1.07

Note: β, standardized effect; SE, standard error; LLCI, lower level confidence interval; ULCI, upper level confidence interval. D1, “Moderate metacognition” vs. “Low metacognition” D2, “High metacognition” vs. “Low metacognition”.

## Data Availability

The original contributions presented in this study are included in the article/Appendix A and Appendix B. Further inquiries can be directed to the corresponding author.

## Appendix A

**Mathematical Metacognition Scale**

Instructions: Please rate the extent to which you agree with each of the following statements regarding your mathematics learning *(Strongly Disagree, Somewhat Disagree, Uncertain, Somewhat Agree, Strongly Agree*).

**I. Metacognitive Knowledge**

*Knowledge of Person*

1 I can accurately evaluate my current mathematics learning status and clearly judge my relative standing in the class.

2 I can correctly identify gaps in my knowledge of specific high school math modules (e.g., I cannot clearly articulate the concept of skew lines or distinguish between a proposition’s negation and its inverse).

3 Provided I pay attention in class, I am confident that I can understand the most difficult problems explained by the teacher.

4 I clearly understand my aptitude for learning different types of mathematical knowledge (e.g., whether I excel in trigonometry versus spatial geometry).

5 I believe I can master the mathematical knowledge taught in class, including concepts, formulas, and theorems.

*Knowledge of Task*

6 When reading a math problem, I have a clear understanding of the given conditions and the unknowns.

7 I have a clear understanding of basic problem types (e.g., open-ended, algebraic, geometric).

8 I believe that understanding mathematical concepts, theorems, and formulas is more conducive to memory and application than rote memorization.

9 I have a clear, holistic grasp of the connections between the mathematical concepts, theorems, and methods I have learned.

*Knowledge of Strategy*

10 I am confident in the effectiveness of the problem-solving methods I employ.

11 I can flexibly apply common mathematical problem-solving methods (e.g., mathematical induction, substitution method, proof by contradiction).

12 I have a relatively clear understanding of the advantages and disadvantages of common mathematical problem-solving methods.

13 I cannot clearly grasp the functions and applicable scopes of mathematical problem-solving approaches.

14 I am clear about the usage conditions for various mathematical methods or theorems (e.g., I can accurately determine which types of sequence problems can be solved using the method of subtracting staggered terms).

**II. Metacognitive Experience**

*Cognitive Experience*

4 I can clearly judge whether the mathematical knowledge learned in high school presents difficulties for me.

5 I often connect newly learned mathematical concepts or theorems with similar existing knowledge.

24 During problem-solving, I can sense the speed of my progress and my mental state in overcoming difficulties.

43 Upon seeing certain problems, I immediately get a sense of whether they are easy or difficult.

50 After reading a problem, I can sense whether I am capable of solving it and gauge my level of confidence.

*Affective Experience*

4 I can flexibly apply common mathematical problem-solving methods (e.g., mathematical induction, substitution method, proof by contradiction).

29 Whenever I fail to solve a math problem successfully, I feel frustrated and anxious, leading me to give up.

28 I believe that understanding mathematical concepts, theorems, and formulas is more conducive to memory and application than rote memorization.

45 Before learning new mathematical knowledge, I often lack confidence in myself.

**III. Metacognitive Monitoring**

*Orientation & Planning*

6 When learning different mathematical topics, I set corresponding learning goals (e.g., when learning solid geometry, I aim to improve my spatial visualization ability).

17 Before starting to solve a math problem, I remind myself to first carefully understand the problem statement.

48 When analyzing a problem, I try to envision several possible solutions and choose the best one to execute.

51 When reviewing for exams, I plan my study schedule strategically (e.g., focusing on error-prone or weak areas).

*Organization & Management*

21 After learning a chapter, I summarize the internal connections between concepts to deepen my understanding (e.g., summarizing exponential, logarithmic, and power functions).

37 When solving problems, I organize and interpret information using equations, graphs, or tables (e.g., drawing function graphs to observe properties).

39 When memorizing knowledge, I create knowledge maps or diagrams to understand concepts from different representational perspectives.

52 When solving complex problems, I break them down into smaller, manageable sub-problems.

*Monitoring & Regulation*

7 If I encounter difficulties, I consider special cases or the simplest scenario (e.g., considering f(x)=x for odd function problems).

9 During problem-solving, I constantly remind myself to pay attention to the problem’s *conditions and conclusion.*

13 When stuck, I try to re-solve the problem or switch to a different strategy.

26 I am aware of my emotional state during problem-solving and consciously adjust it to avoid interference.

35 I constantly check my progress against the problem-solving goal to ensure the validity of my method.

42 During math learning, I can concentrate and avoid distraction.

46 If I don’t understand a concept, I analyze a concrete example of it.

47 If I forget a formula during learning, I derive it myself (e.g., trigonometric induction formulas).

*Feedback & Verification*

12 After learning new knowledge, I test my understanding by solving related problems.

16 After solving a problem, I usually verify the answer using a different method.

19 After solving a problem, I revisit the essence of the problem and summarize the key takeaways.

27 After solving, I reflect on the mathematical knowledge and thinking methods used.

30 After a math lesson, I review the key points mentally or in writing, comparing them with the textbook.

*Reflection & Evaluation*

8 After solving a problem, I wonder if there is a better solution.

11 My estimation of my math exam performance is generally consistent with my actual scores.

33 For problems with multiple solutions, I compare the pros and cons of each method.

44 I often review, verify, and reflect on problems I have previously solved.

54 After a period of learning, I evaluate my learning effectiveness through self-assessment.

55 During math study, I frequently reflect on which content I have not yet mastered.

## Appendix B

**1. Shoe size**

People often see the following table while buying shoes online. The first line can be interpreted as the length of the foot, the second line is what we are used to call “shoe size”.

International shoe size (mm)	220	225	230	235	240	245	250	255	260	265
Common use (European size)	34	35	36	37	38	39	40	41	42	43

Please answer the following questions

(1) Find a function model that satisfies the above conditions.

(2) What is the actual length of a foot that wears children’s shoe in customarily called “size 30”?

(3) A basketball player’s foot is 282 mm long. What size shoe should he wear?

**2. Revolving door**

A revolving door consists of three glass doors capable of rotating within a cylindrical space. The inside diameter of this space is 2 m (200 cm). The diameter of this space is 2 m (200 cm), and the three glass doors divide the cylindrical space into three equal smaller spaces. Below is a top view of the revolving door in three different positions (below left). The entrance and exit are of the same size (shown as dotted lines in the right diagram below), and if the entrance and exit are too large, this revolving glass door will not provide an enclosed space in which air can circulate freely, resulting in unnecessary heat loss, as shown in the diagram on the right below.

Q: What is the maximum arc length at the entrance that will make it possible for air never to flow freely between the entrance and exit?

**3. Fuel filling**

Mr. Wang usually drives a Honda 180TURBO, and there is a gas station A near his home, and a gas station B 10 km away from his home, and the price of No. 95 gasoline at both gas stations A and B this week is RMB 7.60/L. He has previously applied for a free membership of gas station B, and can enjoy a discount of 0.4 Yuan less per liter of gasoline. The following chart shows some of the parameters of the Honda 180TURBO. Please answer the following questions: Analyze which gas station should Mr. Wang choose to fill up his car more cost-effectively?

Manufacturer	Dongfeng Honda	Body type	Trim
Class	Compact car	Length (mm)	4649
Time to market	2016.11	Width (mm)	1800
Engine	1.0T	Height (mm)	1416
Intake Form	Turbocharged	Wheelbase (mm)	2700
Maximum horsepower (PS)	125	Front Wheelbase (mm)	1547
Maximum torque (N·m)	173	Rear Wheelbase (mm)	1563
Maximum speed (km/h)	204	Minimum Ground Clearance (mm)	105
Official 0–100 km/h Acceleration (s)	11.6	Weight (kg)	1243
Actual 0–100 km/h Acceleration (s)	-	Door count	4
Actual 100–0 km/h Braking (m)	-	Seat count	5
Actual Fuel Consumption (L/100 km)	-	Fuel tank (L)	47
Fuel Consumption combined by MIIT * (L/100 km)	5.5	Trunk volume (L)	-
MIIT Pure Electric Range (km)	-	Maximum trunk volume (L)	-
Warranty	Three years or 100,000 km	Trunk interior dimensions (mmm)	-
